# Introducing a standardised register for strengthening the inpatient management of newborns and sick children: Implementation research in selected health facilities of Bangladesh

**DOI:** 10.7189/jogh.14.04086

**Published:** 2024-05-17

**Authors:** Shafiqul Ameen, Sabit Saad Shafiq, K M Tanvir, Ashfia Saberin, Goutom Banik, Ehtesham Kabir ANM, Sabina Ashrafee, Palash Kumar Saha, Bushra Amena, Husam Md Shah Alam, Sabbir Ahmed, Md Nurul Khan, Salmun Nahar, Md Taqbir Us Samad Talha, Sadman Sowmik Sarkar, Aniqa Tasnim Hossain, Sabrina Jabeen, Md Ziaul Haque Shaikh, Md Al-Mahmud, Azim Uddin AFM, Anisuddin Ahmed, Mohammod Jobayer Chisti, Muhammad Shariful Islam, Supriya Sarkar, Sheikh Daud Adnan, Shams El Arifeen, Md Jahurul Islam, Ahmed Ehsanur Rahman

**Affiliations:** 1 International Centre for Diarrhoeal Disease Research, Bangladesh (icddr,b), Dhaka, Bangladesh; 2 Directorate General of Health Services (DGHS), Ministry of Health and Family Welfare, Dhaka, Bangladesh; 3 Save the Children, Dhaka, Bangladesh; 4 UNICEF, Dhaka, Bangladesh; 5 Projahnmo Research Foundation, Dhaka, Bangladesh; 6 World Health Organization, Dhaka, Bangladesh; 7 Independent Consultant, Dhaka, Bangladesh

## Abstract

**Background:**

It is imperative to maintain accurate documentation of clinical interventions aimed at enhancing the quality of care for newborns and sick children. The National Newborn Health and IMCI programme of Bangladesh led the development of a standardised register for managing newborns and sick children under five years of age during inpatient care through stakeholder engagement. We aimed to assess the implementation outcomes of the standardised register in the inpatient department.

**Methods:**

We conducted implementation research in two district hospitals and two sub-district hospitals of Kushtia and Dinajpur districts from November 2022 to January 2023 to assess the implementation outcomes of the standardised register. We assessed the following World Health Organization implementation outcome variables: usability, acceptability, adoption (actual use), fidelity (completeness and accuracy), and utility (quality of care) of the register against preset benchmarks. We collected data through structured interviews with health care providers; participant enrolment; and data extraction from inpatient registers and case record forms.

**Results:**

The average usability and acceptability scores among health care providers were 73 (standard deviation (SD) = 14) and 82 (SD = 14) out of 100, respectively. The inpatient register recorded 96% (95% confidence interval (CI) = 95–97) of under-five children who were admitted to the inpatient department (adoption – actual use). The proportions of completed data elements in the inpatient register were above the preset benchmark of 70% for all the assessed data elements except ‘investigation done’ (24%; 95% CI = 23–26) (fidelity – completeness). The percentage agreements between government-appointed nurses posted and study-appointed nurses were above the preset benchmark of 70% for all the reported variables (fidelity – accuracy). The kappa coefficient for the overall level of agreement between these two groups regarding reported variables indicated moderate to substantial agreement. The proportion of newborns with sepsis receiving injectable antibiotics was 62% (95% CI = 47–75) (utility – quality of care). We observed some variability in the completeness and accuracy of the inpatient register by district and facility type.

**Conclusions:**

The inpatient register was positively received by health care providers, with evaluations of implementation outcome variables showing encouraging results. Our findings could inform evidence-based decision-making on the implementation and scale-up of the inpatient register in Bangladesh, as well as other low- and middle-income countries.

Bangladesh has significantly reduced its under-five mortality rate from 48 deaths per thousand live births to 31 deaths per thousand live births between 2011 and 2022 [1–4]. Despite this progress, around 80 000 children under five years of age still die each year in Bangladesh [5]. Most of these deaths are avoidable and occur due to causes such as pneumonia; serious infections; prematurity and low birth weight; and intrapartum-related complications, with nearly two-thirds occurring within the first 28 days of life [4]. To prevent these deaths, it is important to improve the quality of care provided to newborns and sick children in hospital settings.

Improving documentation practices by strengthening the patient record system and documentation flow can significantly improve quality of care in health facilities, as it can enable effective disease monitoring, the exploration of health care delivery patterns, and the generation of practical evidence of the impact of medical care and service delivery models for children [6–8]. A comprehensive health systems approach which would include the timely provision of quality inpatient care, the identification of individuals at high risk, and the recording and reporting clinical data can save many lives and prevent morbidity [9–11].

Bangladesh has scaled up highly effective evidence-based interventions targeting major causes of under-five deaths. These include immediate newborn care and helping babies breathe for birth asphyxia; the administration of antenatal corticosteroid for suspected preterm labour; kangaroo mother care for preterm and low birth-weight babies; the administration of injectable antibiotics for serious infections, and the establishment of special care newborn units (SCANUs) for critically ill newborns [12–15]. The country has also introduced standardised service registers in corners related to newborn and child care targeting these interventions, such as postnatal care corners, integrated management of childhood illness (IMCI) corners; labour wards and operation theatres; SCANUs; and kangaroo mother care corners. Monthly reports are generated from these service registers and are submitted to routine health information systems. However, there is no dedicated service register or monthly reporting form in the paediatric inpatient department, where a substantial number newborns and sick children are managed every year.

Acknowledging this, the National Newborn Health and IMCI programme (NNHP & IMCI programme) of the Directorate General of Health Services took the initiative to develop and introduce a standardised register for managing newborns and sick children during inpatient care. It also decided to implement this standardised register in select districts and to conduct research to assess implementation outcomes before national scale up. In response, we wanted to assess the implementation outcomes of the standardised register for inpatient management of newborns and sick children in select districts to inform evidence-based scale up in Bangladesh.

## METHODS

### Development and implementation of inpatient register

The NNHP & IMCI programme led the design and development of this register, along with an implementation plan through stakeholder engagement. This register was rolled out in multiple districts across Bangladesh with technical assistance from development partners through four steps – the development process; training; district implementation; and monitoring and supervision.

#### Development process

The NNHP & IMCI programme established a technical committee to develop an inpatient register for newborns and sick children with members from government departments under the Directorate General of Health Services and in coloration with development partners. This committee investigated documentation practices in district and sub-district hospitals, particularly within the paediatric inpatient department, where they collected data from case record forms and registers to gain insights on how paediatric patients were being managed. They also reviewed various national and global guidelines and strategies, as well as registers and case record forms available worldwide. The committee examined similar inpatient registers used within Bangladesh, such as the kangaroo mother care register and the SCANU register. Afterwards, four consultative workshops were organised to develop the inpatient register, which was structured in such a way that the details of one patient can be documented on a single page. Each page is divided into two rows, with the first row to be completed upon admission and the second to be filled out at the time of discharge (Table S1 of the **Online Supplementary Document**). Nurses working in the inpatient departments are responsible for filling up this register based on the information recorded in the individual patient case record forms. The technical committee also developed a monthly reporting form based on the register which participating facilities are meant to complete every month and send to the NNHP & IMCI programme.

#### Training

In October 2022, the NNHP & IMCI programme organised four training sessions for master trainers in Dhaka. Each facility had at least two trained master trainers who subsequently conducted training for the above-mentioned nurses.

#### District implementation

The inpatient register was implemented in five districts between November 2022 and January 2023, with technical assistance from icddr,b, Save the Children, the United Nations Children’s Fund (UNICEF), and the World Health Organization (WHO). Forty health facilities were involved, including one medical college hospital, four district hospitals, and 35 sub-district hospitals.

#### Monitoring and supervision

The NNHP & IMCI programme and health managers at district and sub-district levels, along with the master trainers, were jointly responsible for monitoring and supervising the register’s implementation. The challenges of implementing the register and insights gained from monthly reporting forms were discussed during monthly meetings at participating facilities. Monthly reporting forms were sent to the NNHP & IMCI programme, based on which online meetings were organised to assess and review progress.

### Study design

The icddr,b carried out an implementation research to assess the implementation outcomes of the standardised register in two districts in Bangladesh. The research itself was designed as a cross-sectional study. Based on the decision of the technical committee, the implementation outcomes were adapted from the framework for implementation research developed by the WHO [16]. The primary implementation outcomes were the usability, acceptability, adoption (actual use), fidelity (completeness and accuracy), and utility (quality of care) of the register. The technical committee set a benchmark to successfully demonstrate each outcome variable. The primary research questions are detailed in **Table 1**.

**Table 1:** Primary research questions along with benchmark set by technical committee to successfully demonstrate each outcome variable based on framework for implementation research developed by WHO

WHO’s framework	Research questions	Proposed indicator	Benchmark
Usability	What is the usability of the inpatient register among health care provider?	Average score using the SUS	>70
Acceptability	What is the acceptability of the inpatient register among health care provider?	Average score using the TAM	>80
Adoption	Actual use: do health care providers use the inpatient register?	Proportion of admitted newborn and children documented in the inpatient register	>80%
Fidelity	Completeness: do health care providers fill up ‘investigation done’ column of the inpatient register?	Proportion of completed ‘investigation done’ column among newborn and children recorded in the inpatient register	>70%
	Completeness: do health care providers fill up ‘care received during admission’ column in the inpatient register?	Proportion of completed ‘care received during admission’ column among newborn and children recorded in the inpatient register	>70%
	Completeness: do health care providers fill up ‘drugs received during admission’ column in the inpatient register?	Proportion of completed ‘drugs received during admission’ column among newborn and children recorded in the inpatient register	>70%
	Completeness: do health care providers fill up ‘final diagnosis’ column in the inpatient register?	Proportion of completed ‘final diagnosis’ column among newborn and children recorded in the inpatient register	>70%
	Completeness: do health care providers fill up ‘outcome of treatment’ column in the inpatient register?	Proportion of completed ‘outcome of treatment’ column among newborn and children recorded in the inpatient register	>70%
	Accuracy: what is the level of observed agreement between government-appointed nurses and study-appointed nurses for ‘oxygen given’ variable in the inpatient register?	Level of observed agreement between government-appointed nurses and study-appointed nurses regarding recording ‘oxygen given’ variable in the inpatient register	>70%
	Accuracy: what is the level of observed agreement between government-appointed nurses and study-appointed nurses for ‘injectable gentamicin’ variable in the inpatient register?	Level of observed agreement between government-appointed nurses and study-appointed nurses regarding recording ‘injectable gentamicin’ variable in the inpatient register	>70%
	Accuracy: what is the level of observed agreement between government-appointed nurses and study-appointed nurses for ‘diagnosis – severe pneumonia’ variable in the inpatient register?	Level of observed agreement between government-appointed nurses and study-appointed nurses regarding recording ‘diagnosis – severe pneumonia variable in the inpatient register	>70%
	Accuracy: what is the level of observed agreement between government-appointed nurses and study-appointed nurses for ‘refer’ variable in the inpatient register?	Level of observed agreement between government-appointed nurses and study-appointed nurses regarding recording ‘refer’ variable in the inpatient register	>70%
Utility	Quality of care: do the admitted children receive injectable antibiotic for newborn sepsis?	Proportion of admitted children receiving injectable antibiotic for newborn sepsis	>70%

TAM – technology acceptance model, SUS – system usability scale

### Study settings

We carried out the study in the Kushtia and Dinajpur districts of Bangladesh due to them having similar newborn and under-five mortality rates to the national estimates (Figure S1 of the **Online Supplementary Document**). The Kushtia district, situated in the Khulna division of western Bangladesh, has a population of more than two million. The Dinajpur district, located in the Rangpur division of northern Bangladesh, has around three and a half million inhabitants. Both districts primarily consist of rural areas [17]. These districts have well-functioning inpatient and outpatient care for newborns and sick children, owing to several maternal, newborn, and child health initiatives undertaken by the government. One district hospital and one sub-district hospital (also known as a *upazila* health complex) were selected from each district in consultation with the NNHP & IMCI programme and district health managers (civil surgeons). These were the Kumarkhali sub-district hospital from the Kushtia district and Hakimpur sub-district hospital from the Dinajpur district, which serve as the first referral facility for inpatient management of newborns and sick children. In turn, the Kushtia and Dinajpur district hospitals serve as the secondary referral facility covering the whole district.

### Study participants

We included children aged between 0 and 59 months who had been admitted to the paediatric indoor department of the participating health facilities, as well as government-employed nurses who worked in the paediatric indoor department and had received training on the inpatient register.

### Sample size and sampling

We calculated the sample size based on the preset benchmark so as to successfully answer each primary research question. Assuming a 5% margin of error, 95% confidence interval (CI), and 5% non-response rate, the highest adjusted sample size for the adoption, fidelity, and utility indicators was 340 children aged between 0 and 59 months who were admitted to the paediatric indoor department per facility per month. Therefore, the total sample size was 4080 children. We approached all the children admitted to the inpatient department of the participating health facilities for enrolment.

Assuming a 10% margin of error, 95% confidence interval, and 10% non-response rate, the highest adjusted sample size for the usability and acceptability indicators was 90 government-employed nurses who had worked in the paediatric indoor department and had received training on the inpatient register. We approached all such nurses in the Kushtia and Dinajpur districts for enrolment.

### Data collection

We carried out our study during a three-month period from 1 November 2022 to 31 January 2023. We collected the data using structured interview of the health care providers; participant enrolment; and data extraction from inpatient register and case record forms. Prior to finalisation, all data collection tools, including a custom-designed Android application, underwent separate field testing with a minimum of five participants. We obtained administrative approvals from the NNHP & IMCI programme, including for the patient enrolment and data extraction from the inpatient registers and the case record forms.

#### Structured interviews

Using adapted versions of the System Usability Scale (SUS) [18] and the Technology Acceptance Model (TAM) tool [19], we conducted structured interviews with nurses who had received training on the paediatric inpatient register in the Kushtia and Dinajpur districts to assess the usability and acceptability of the inpatient register among health care providers. The SUS, a dependable tool for evaluating the usability of systems, products, or services, comprises a 10-item questionnaire featuring five response options, ranging from ‘strongly agree’ to ‘strongly disagree.’ SUS scores range from 0 to 100 and provide insights into overall user satisfaction and usability [18]. The TAM, meanwhile, is a widely recognised psychological model used to understand how health care providers perceive and embrace new information technology or systems [19]. We obtained written informed consents from the health care providers prior to the structured interview.

#### Participant enrolment

Using a structured participant enrolment tool, non-medical study staffs enrolled patients by collecting basic demographic information (name, age, gender, admission date and time, and registration number) for all admitted children in the paediatric inpatient department at the time of admission throughout the three-month study. We also obtained written informed consents from the mother/caregivers of the children at the time of enrolment.

#### Data extraction from inpatient register

Trained data collectors employed a structured data extraction tool to retrieve information from the inpatient register for children participating in the study and had subsequently been discharged from the hospital. A clinical supervisor oversaw the data extraction process, including the re-extraction of 5% of the observations.

#### Data extraction from case record forms

Trained study nurses extracted data from the case record forms for children whose information was obtained from the inpatient register using a specially designed data extraction tool similar to the inpatient register. We ensured that individuals involved in patient enrolment and data extraction from the case record forms had no access to the inpatient register's information. The same clinical supervisor monitored the case record form data extraction and performed 5% re-extraction, and provided feedback as required.

### Data analysis

We assessed the inpatient register’s primary outcomes, including usability, acceptability, adoption, fidelity, and utility. We measured usability through the health care providers’ perception of the register’s usability and acceptability through their acceptance of the register. Moreover, we measured adoption by comparing the number of admitted children from patient enrolment with the children documented in the inpatient register. We measured fidelity – completeness by the completion of data elements such as investigation done, care received during admission, drugs received during admission, final diagnosis, and treatment outcome among children recorded in the inpatient register, and fidelity – accuracy through the percentage agreement on reporting variables (oxygen given; injectable gentamicin; diagnosis – severe pneumonia; and outcome of treatment – refer) between government-appointed nurses in the inpatient department and study-appointed nurses. Lastly, we measured utility through the reporting proportion of newborns with sepsis receiving injectable antibiotics.

We presented usability and acceptability as means with standard deviations (SDs) disaggregated by health care provider age, facility type, and district. We used independent *t*-tests to determine whether the difference of means between categories was significant. We presented adoption, fidelity, and utility as percentages with 95% CIs for patient’s age, sex, facility type, district, and month of assessment. We used proportion tests to determine whether the difference of percentage between sex, facility type, and district was significant. We also used analysis of variance (ANOVA) to check the difference for the categories of month of assessment and patient’s age. The statistical significance was reported at a 5% level of significance.

We also presented the agreement between government-appointed nurses and study-appointed nurses as a kappa (κ) coefficient to report accuracy, where agreement is interpreted per the following categories: poor (κ≤0.00), slight (κ = 0.01–0.19), fair (κ = 0.20–0.39), moderate (κ = 0.40–0.59), substantial (κ = 0.60–0.79), and perfect (κ≥0.80) [20]. Data analysis was conducted using Stata version 15.0 [21].

### Ethical considerations

We received ethical approval (PR-21112) from the Institutional Review Board of the icddr,b. We also obtained informed written consent from the mother/caregiver of the children and the health care providers, and administrative approval from the NNHP & IMCI programme.

## RESULTS

**Figure 1:**
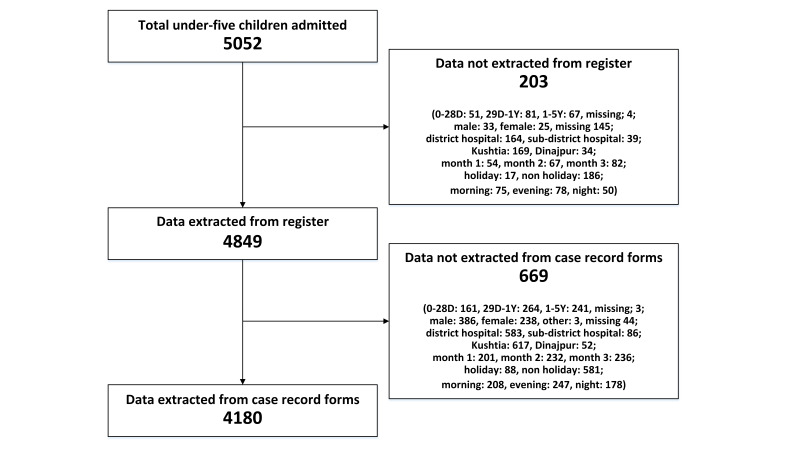
Sample size for assessments.

In total, 5052 under-five children were admitted in the paediatric inpatient department between 1 November 2022 and 31 January 2023. We extracted data from the inpatient register for 4849 (96%) children and from case record forms for 4180 (83%) children (**Figure 1**).

For the inpatient register data, 2102 (43%) children were aged between 29 days and 11 months, 2770 (61%) were male, 4011 (83%) were from the Kushtia district, and 3909 (81%) were from district hospitals. Moreover, 2069 (52%) were discharged with advice, while 36 (1%) children died while hospital stay (**Table 2**).

**Table 2:** Background characteristics of the participants according to data extracted from inpatient register (N = 4849)

Characteristics	n (%)
**Age**	
0–28 days	1210 (25.0)
29 days to 11 months	2102 (43.4)
12 to 59 months	1532 (31.6)
Missing	5
**Sex**	
Male	2770 (60.6)
Female	1795 (39.3)
Others	4 (0.1)
Missing	280
**District**	
Kushtia	4011 (82.7)
Dinajpur	838 (17.3)
Missing	0
**Facility type**	
District hospital	3909 (80.6)
Sub-district hospital	940 (19.4)
Missing	0
**Month of assessment**	
Month 1	1517 (31.3)
Month 2	1705 (35.2)
Month 3	1627 (33.6)
Missing	0
**Outcome of treatment**	
Discharge with advice	2069 (52.1)
Discharge on request	829 (20.9)
DORB	78 (2.0)
Refer	442 (11.1)
Absconded	517 (13.0)
Death	36 (0.9)
Missing	878
**Total**	4849

DORB – discharge on risk bond

We enrolled 176 health care providers from the Kushtia and Dinajpur districts who had received training on inpatient register. All of them were nurses; 94 (53%) less than 35 years old, 111 (63%) were from the Dinajpur district, and 149 (85%) were from sub-district hospitals (Table S2 of the **Online Supplementary Document**).

**Figure 2:**
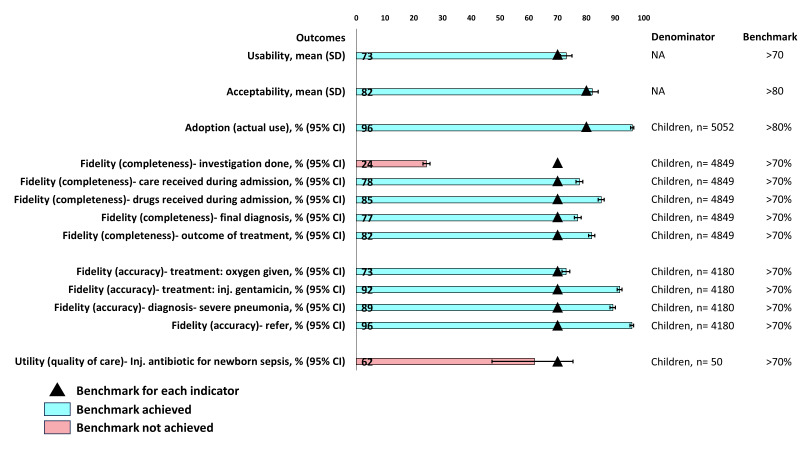
Assessments of WHO implementation research outcomes for inpatient register, presented as means with SDs for usability and acceptability indicators and percentages with 95% CIs for adoption, fidelity, and utility indicators (utility and acceptability: n = 176; adoption: n = 5052; fidelity-completeness and utility-outcome: n = 4849; fidelity-accuracy: n = 4180; utility-quality of care: n = 50).

For the assessments of the WHO implementation research outcomes for inpatient registers (**Figure 2**), the average usability score among health care providers according to the SUS was 73 (SD = 14) out of 100, while average acceptability score per the TAM was 82 (SD = 14) out of 100. The inpatient register recorded 96% (95% CI = 95–97) of under-five children who were admitted in the paediatric inpatient department (adoption- actual use). The proportions of completed data elements in the inpatient register were above the preset benchmark of 70% for all the assessed data elements, except for ‘investigation done’ (24%; 95% CI = 23–26) (fidelity – completeness). The percentage agreement between government-appointed nurses and study-appointed nurses were above the preset benchmark of 70% for all the reported variables (fidelity – accuracy), with the κ coefficient for the overall level of agreement between these two groups indicating moderate to substantial agreement (Table S3 of the **Online Supplementary Document**). However, the proportion of newborns with sepsis receiving injectable antibiotics according to data extracted from the inpatient register was 62% (95% CI = 47–75), which was below the preset benchmark of 70% (utility – quality of care).

In view of the influence of various factors on the implementation research outcomes for inpatient register, the mean SUS and TAM scores were significantly higher in the Dinajpur district, while the completeness and accuracy of the majority of the reported data elements were higher in the sub-district hospitals and the Dinajpur district (**Table 3**).

**Table 3:** Influence of different factors on implementation research outcomes for inpatient register*

		Patient-related factors					Provider-related factors		Facility-related factors						
		**Age**			**Sex**		**Age**		**Facility type**		**District**		**Month of assessments**		
		**0–28 days**	**29 d to 11 months**	**12–59 months**	**Male**	**Female**	**<35 years**	**≥35 years**	**District hospital**	**Sub-district hospital**	**Kushtia**	**Dinajpur**	**Month 1**	**Month 2**	**Month 3**
**Usability**	**x̄ (SD)**						74(15)	72(12)	75 (19)	73 (13)	64 (16)	78 (9)			
	***P*-value**						0.088		0.221		<0.001				
**Acceptability**	**x̄ (SD)**						83(14)	81(13)	72 (21)	84 (11)	69 (11)	90 (7)			
	***P*-value**						0.169		0.005		<0.001				
**Adoption (actual use)**	**% (95% CI)**	96 (95–97)	96 (95–97)	96 (95–97)	99 (98–99)	99 (98–99)			96 (95–97)	96 (94–97)	96 (95–97)	96 (95–97)	97 (95–97)	96 (95–97)	95 (94–96)
	***P*-value**	0.256			0.675				0.669		0.838		0.287		
**Fidelity (completeness) investigation done**	**% (95% CI)**	24 (22–27)	28 (26–30)	20 (18–22)	25 (23–26)	24 (22–26)			30 (28–31)	2 (1–3)	28 (27–29)	8 (6–10)	23 (21–26)	27 (25–29)	23 (21–25)
	***P*-value**	<0.001			0.894				<0.001		<0.001		<0.001		
**Fidelity (completeness) – care received during admission**	**% (95% CI)**	80 (78–82)	79 (77–81)	73 (71–76)	77 (75–78)	79 (77–80)			78 (77–79)	75 (72–78)	75 (73–77)	80 (78–82)	75 (73–77)	80 (78–82)	77 (75–79)
	***P*-value**	<0.001			0.203				0.041		<0.001		0.006		
**Fidelity (completeness) – drugs received during admission**	**% (95% CI)**	86 (84–88)	88 (87–89)	80 (78–82)	85 (84–87)	85 (83–87)			84 (83–85)	89 (87–91)	85 (84–86)	85 (82–87)	84 (82–86)	85 (83–86)	87 (85–88)
	***P*-value**	<0.001			0.697				<0.001		0.838		0.113		
**Fidelity (completeness) – final diagnosis**	**% (95% CI)**	73 (71–77)	79 (77–81)	77 (75–79)	77 (75–78)	79 (77–80)			76 (74–77)	83 (80–85)	75 (73–76)	88 (86–90)	73 (71–76)	81 (79–83)	76 (74–79)
	***P*-value**	<0.001			0.125				<0.001		<0.001		0.915		
**Fidelity (completeness) – outcome of treatment**	**% (95% CI)**	78 (75–80)	82 (84–86)	83 (81–84)	81 (80–83)	83 (81–85)			79 (78–81)	93 (91–94)	81 (79–82)	87 (85–90)	80 (78–82)	83 (81–85)	82 (80–84)
	***P*-value**	<0.001			0.189				<0.001		<0.001		0.117		
**Fidelity (accuracy) – treatment oxygen given**	**% (95% CI)**	72 (69–74)	69 (67–71)	79 (77–81)	72 (70–74)	73 (71–76)			68 (66–70)	92 (90–94)	73 (72–75)	72 (69–75)	68 (66–71)	77 (75–79)	73 (71–76)
	***P*-value**	<0.001			0.405				<0.001		0.618		<0.001		
**Fidelity (accuracy) – treatment injectable gentamicin**	**% (95% CI)**	89 (87–91)	90 (89–92)	96 (94–97)	92 (90–93)	92 (90–93)			91 (90–92)	92 (90–94)	90 (89–91)	99 (99–100)	90 (88–92)	92 (90–93)	93 (92–94)
	***P*-value**	<0.001			0.869				0.638		<0.001		0.019		
**Fidelity (accuracy) – diagnosis – severe pneumonia**	**% (95% CI)**	97 (95–98)	84 (82–85)	91 (89–93)	89 (88–90)	90 (88–91)			88 (87–89)	93 (91–95)	88 (89–90)	90 (87–92)	88 (87–90)	89 (87–91)	90 (88–92)
	***P*-value**	<0.001			0.631				<0.001		0.549		0.436		
**Fidelity (accuracy) – refer**	**% (95% CI)**	90 (88–92)	98 (97–98)	98 (97–99)	89 (88–90)	90 (88–91)			95 (94–96)	99 (98–100)	95 (94–96)	99 (98–99)	96 (95–97)	95 (94–96)	96 (95–97)
	***P*-value**	<0.001			0.096				<0.001		<0.001		.0.552		
**Utility (quality of care) – injectable antibiotic for newborn sepsis**	**% (95% CI)**	50 (34–66)	29 (4–71)	33 (1–91)	44 (24–65)	43 (23–66)			57 (39–73)	15 (2–45)	59 (41–75)	19 (4–46)	38 (20–59)	50 (26–74)	67 (22–96)
	***P*-value**	0.537			0.972				0.009		0.007		0.435		

CI – confidence interval, SD – standard deviation, x̄ – mean

*Utility and acceptability: n = 176; adoption: n = 5052; fidelity-completeness and utility-outcome: n = 4849; fidelity-accuracy: n = 4180; and utility-quality of care: n = 50. *P*-values <0.05 indicate a statistically significant difference in mean or proportion.

## DISCUSSION

The introduction of standardised clinical registers has the potential to improve the quality of care and outcomes of patients admitted in the hospitals [22-24]. Here we reported on our assessment of WHO implementation outcome variables (usability, acceptability, adoption, fidelity, and utility [16]) for the implementation of a standardised register for the inpatient management of newborns and sick children. We found that health care providers accepted the inpatient register and found it usable; that it universally captured all eligible newborns and sick children admitted to the inpatient department; and that its data elements were mostly completed. The recording of variables in the inpatient register by health care providers showed moderate to substantial agreement with the recording done by study-recruited nurses. We set a benchmark prior to conducting this implementation research, this might have created a sense of accountability among the health care providers, which may have contributed to our positive findings.

The WHO has recommended the judicious and timely administration of injectable antibiotics for newborns admitted to the hospital with serious infections [25], as well as the administration of the first dose of injectable antibiotics and urgent referral for newborns visiting outpatient departments with suspected severe bacterial infections [26]. The Every Newborn Action Plan identified comprehensive health indicators used for monitoring newborn health progress effectively [27]. Population-based surveys such as Demographic and Health Surveys and Multiple Indicator Cluster Surveys are not suitable for collecting information on the coverage of treatment of newborn infections with injectable antibiotics, mostly due to issues with maternal recall error and bias [28–30]. This makes routine health information systems the most appropriate way to measure coverage of treatment of newborn infections with injectable antibiotics. In Bangladesh, IMCI was introduced nationally by the government in the early 1990s for the outdoor management of childhood illnesses, as recommended by the WHO for low- and middle-income countries [31]. Data on the management of newborn infections with injectable antibiotics has also been available in routine health information systems for outdoor management in Bangladesh. However, it is not possible to track this coverage indicator for the inpatient department, except for those newborns who are admitted to SCANUs, where structured registers and monthly reporting to routine health information systems are available. The inpatient register for newborns and sick children presents a unique opportunity to track the treatment of newborn infections with injectable antibiotics. However, we observed that only 62% of newborns admitted to the inpatient department with sepsis received injectable antibiotics. Three possible explanations exist for this low coverage: either the intervention was not performed; or it was performed, but not documented in the case record forms; or it was performed and documented in the case record forms, but not in the inpatient register. Exploring whether injectable antibiotics were administered or not was beyond the scope of this study. However, we identified issues with the design of the case record forms used to fill up the inpatient register, which might have contributed to the low coverage of injectable antibiotic treatment for newborn infections. In Bangladesh, unstructured case record forms are used in public hospital inpatient departments, resulting in inconsistency in documentation practices [30,32]. This also likely contributed to the low proportion of completion for the ‘investigations done’ data element in the inpatient register in our study. This is a consequence of the prevalent practice in Bangladesh, where laboratory investigation reports are typically handed over to patients at the time of discharge, along with the discharge certificate, leading to the documentation of investigation data in case record forms being inadequately maintained. Therefore, we recommend introducing structured case record forms in hospitals to address this issue. Regular monitoring and supervision from central and district-level health managers are essential in identifying reasons for the low coverage of injectable antibiotic treatment for newborn infections and the low proportion of completion of the ‘investigations done’ data element in the inpatient register.

We also assessed the completeness and accuracy of the inpatient register to determine the extent to which the intervention was executed in accordance with its intended design and objectives [16] and found some variability in both variables. This is consistent with several studies that reported similar issues with health registers [33–40]. Moreover, sub-district hospitals in our study had higher rates of completeness and accuracy than district hospitals, which may be due to differences in caseload between these two types of health facilities. Specifically, district hospitals serve as referral facilities for a district and provide services to patients from all over the district, while sub-district hospitals serve as primary-level health facilities with lower caseloads than district hospitals. These differences can also explain the higher rate of completeness and accuracy in the Dinajpur district than the Kushtia district. Notably, the Dinajpur district has a separate medical college hospital that serves as the highest-level referral facility, resulting in comparatively lower caseloads in other public health facilities in the Dinajpur district. Conversely, caseloads are higher in the district hospital of the Kushtia district as there was no functioning medical college hospital during our study period. Several studies have reported that poor documentation practice by health care providers is associated with higher caseloads [41–44]. To improve the completeness and accuracy of the register, dedicated staff can be appointed in facilities with higher caseloads for documentation in the inpatient register. Different data quality improvement initiatives (including routine audits by facility-, district-, and national-level health managers) and on-site mentoring have also proved effective [45,46]. Shifting towards e-registers can be a viable solution, although managing them alongside large databases can be challenging in low-resource settings. This challenge must be taken into account when considering the feasibility of implementing e-registers in such settings [47,48].

### Strengths and limitations

Our study had several strengths. First, we designed this study based on the framework for implementation research developed by the WHO and reported most of the implementation outcomes. Second, we selected two high-burden facilities (the Kushtia district hospital and the Kumarkhali sub-district hospital) and two low-burden facilities (the Dinajpur district hospital and the Hakimpur sub-district hospital) to assess the effect of caseload on outcome variables. Lastly, we collected data from trained nurses using a custom-designed Android application to optimise data management.

However, our study also has some limitations. First, we did not choose the implementation districts and health facilities randomly, meaning our results cannot be generalised to the whole country due to selection bias. The Kushtia and Dinajpur districts predominantly have plain land, while other parts of the country with exposed coastal areas, hills, floods, and extreme drought zones might face different challenges in providing health care in the facilities, which we could not explore in this study. We also acknowledge that the implementing facilities are relatively better functioning because of other health initiatives taken in the Kushtia and Dinajpur districts over the past decade. Despite accounting for case mix and hospital differences, we observed limited variability in the background and clinical profiles of patients admitted to both district and sub-district hospitals in Bangladesh, making our study findings applicable to most parts of Bangladesh. Second, the presence of study staff in the inpatient department may have introduced the Hawthorne effect [49] in the performance of the health care providers in filling up the inpatient register. To mitigate this, we established rapport between health care providers and the data collection team through sustained interactions by conducting preparatory workshops at each facility. Third, we only extracted data from the inpatient register and case record forms, but did not explore whether care was actually provided to the patients by the health care providers. This prevented us from investigating whether an intervention was not given or not documented in the case record forms by looking at the data. Fourth, we did not explore other WHO implementation outcome variables such as implementation cost and sustainability due to time and resource limitations. We recommend conducting future studies to explore the cost-effectiveness of introducing an inpatient register in health facilities and post-implementation follow-ups to determine the sustainability of such interventions Lastly, we reported the accuracy of health care providers in filling up the inpatient register by comparing it with the performance of study-recruited nurses. We acknowledge that study-recruited nurses may not be considered the true gold standard for measuring the validity and accuracy of the inpatient register filled up by health care providers. We therefore reported percentage agreement and κ coefficient instead of diagnostic accuracy tests such as sensitivity, specificity, positive and negative predictive values, and area under the curve, which require such gold standards.

## CONCLUSIONS

Healthcare providers have shown a positive attitude towards the register, while our assessment of implementation outcomes has shown promising results. This may be attributed to the extensive stakeholder engagement process led by the Government of Bangladesh from the development of the inpatient register to its implementation and assessments. However, we also found low proportions in some of the indicators and variability in the completeness and accuracy of the inpatient register by district and type of facility, mostly due to the differences in caseload. The experience acquired from this demonstration, coupled with the insights derived from this implementation research, can contribute to the formulation of data-driven decisions relating to the implementation and scale-up of a standardised register for inpatient management of newborns and sick children in Bangladesh and other low- and middle-income countries.

## Additional material


Online Supplementary Document
