# Supplementary material for: Introducing a standardised register for strengthening the inpatient management of newborns and sick children: Implementation research in selected health facilities of Bangladesh
Source: J Glob Health. 2024 May 17;14:04086. doi: 10.7189/jogh.14.04086 (PMC11097124; doi:10.7189/jogh.14.04086)
Supplement: Online Supplementary Document [file jogh-14-04086-s001.pdf]

Title: Introducing Standardised Register for Strengthening the Inpatient Management of Newborns and Sick Children: Implementation Research in Selected Health Facilities of Bangladesh

Online Supplementary Document

Supplementary Figure S1: Study sites

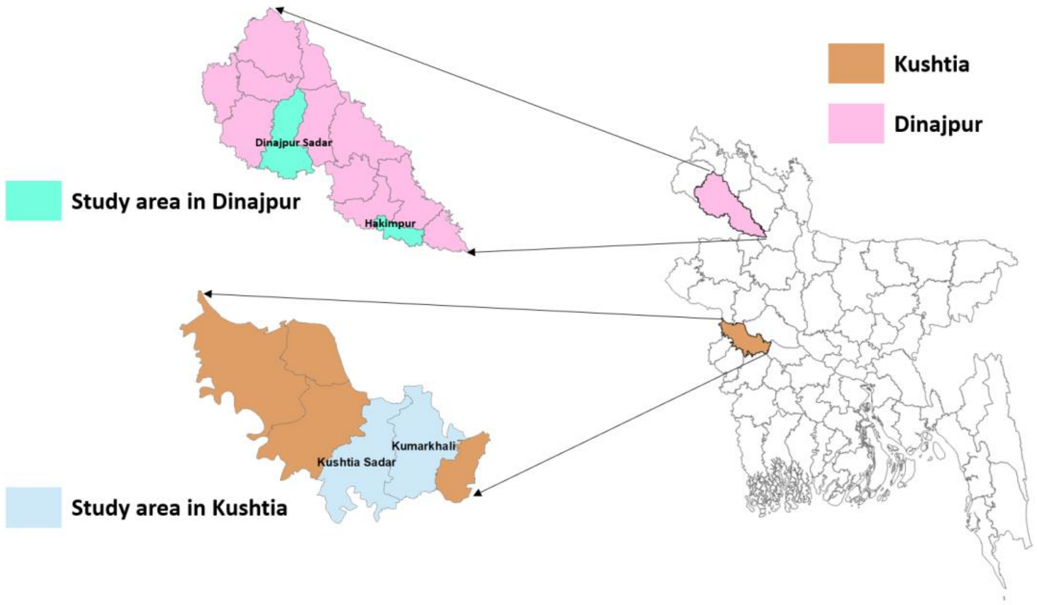

Supplementary Table S1: Inpatient register for newborns and sick children

(Columns 1-8 to be filled during admission & 9-14 after discharge to be filled at the time of discharge)

Serial No	Registration No	Date & Time of Admission in Pediatric department	The place where the child first came in this facility	Child's identity, address and parent's mobile phone number	Information on referral in this facility	Physical examination at admission	Danger sign present at admission	
1	2	3	4	5	6	7	8	
			☐ IMCI Corner/Outdoor<br><br>☐ Emergency	Name of the child:<br>Age: .... Year .... Month .... Day<br>Gender: ☐ Male ☐ Female<br>☐ Other gender<br>Mothers Name:<br>Fathers Name:<br>Mobile Number:<br>Address:<br>• House/Holding no:<br>• Village/Moholla:<br>• Union/Ward:<br>• Upazila/Municipality/City Corporation:<br>• District:	Was the child referred to this health facility?<br>☐ Yes ☐ No<br><br>From where was the child referred?<br>☐ Other units of the same health facility<br>☐ Govt medical college hospital/ Specialized hospital<br>☐ District hospital<br>☐ MCWC<br>☐ Upazila health complex<br>☐ UHC<br>☐ Community Clinic<br>☐ Private hospital /Clinic/ Chamber<br>☐ NGO hospital /Clinic<br>☐ Others (Specify):<br>...	Weight: .... Kilogram<br>Height: .... cm<br>Temperature: .... ☐ °C ☐ °F<br>Respiration Rate: .... /min<br>Heart Rate: .... /min<br>Oxygen Saturation/SpO_2 .... %<br>MUAC: .... cm<br><br>Stridor: ☐ PRESENT ☐ ABSENT<br>Edema: ☐ PRESENT ☐ ABSENT<br>Anemia: ☐ PRESENT ☐ ABSENT<br>Dehydration: ☐ PRESENT ☐ ABSENT<br>Visible Birth defect: ☐ PRESENT ☐ ABSENT<br><br>☐ Others (Specify):<br>...	**0-59 days**<br>☐ Not feeding well<br>☐ Convulsion/ history of convulsions<br>☐ Drowsy or unconscious<br>☐ Movement only when stimulated or no movement at all<br>☐ Fast breathing<br>☐ Grunting<br>☐ Severe chest indrawing<br>☐ Hypothermia (< 35.5°C or < 96°F)<br>☐ Hyperthermia (≥ 37.5°C or ≥ 99.5°F)<br>☐ Central cyanosis<br>☐ Bulging fontanels<br>☐ Apnoea<br>☐ Persistent Vomiting	**2-59 months**<br>☐ Unable to drink or breastfeed<br>☐ Convulsion/ history of convulsions<br>☐ Lethargic or unconscious<br>☐ Vomits everything

Investigation done	Care received during admission	Drugs received during admission		Final diagnosis with ICD 10 code during discharge		Outcome of treatment	Comments & signature
9	10	11		12		13	14
☐ CBC<br>☐ S. Electrolytes<br>☐ C Reactive protein<br>☐ Blood sugar<br>☐ Blood grouping and Rh Typing<br>☐ S. Bilirubin<br>☐ Blood C/S<br>☐ Urine C/S<br>☐ Urine RME<br>☐ Chest X-ray<br>☐ CSF study<br>☐ Stool R/E<br>☐ Others (Specify):<br>................................<br>................................	☐ Thermal care<br>☐ Oxygen<br>☐ Cup feeding<br>☐ NG feeding<br>☐ IV fluid<br>☐ Phototherapy<br>☐ Oral antibiotic<br>☐ Injectable antibiotic<br>☐ Blood transfusion<br>☐ Nebulization<br>☐ Therapeutic milk (F75/F100)<br>☐ Catheter<br>☐ Others (Specify):<br><br>..................................	☐ Inj. Ampicillin<br>Date from .... To ....<br><br>☐ Inj. Ceftriaxone<br>Date from .... To ....<br><br>☐ Name of the Medicine:<br>Date from .... To ....<br><br>☐ Name of the Medicine:<br>Date from .... To	☐ Inj. Gentamicin<br>Date from .... To ....<br><br>☐ Name of the Medicine:<br>Date from .... To ....<br><br>☐ Name of the Medicine:<br>Date from .... To ....<br><br>☐ Name of the Medicine:<br>Date from .... To	☐ Newborn sepsis P36.9<br>☐ Septicaemia of older children A41.9<br>☐ Diarrhoea with severe dehydration A09<br>☐ Drowning W74<br>☐ Severe Pneumonia J18.9<br>☐ Bronchiolitis J21<br>☐ Bronchial asthma J45<br>☐ Respiratory tract infections J98.7<br>☐ Birth asphyxia P21<br>☐ Anaemia D64.9	☐ Severe Acute Malnutrition (SAM) E46<br>☐ Dengue A97.9<br>☐ Thalassaemia D56<br>☐ Convulsion of newborn P90<br>☐ Febrile convulsions R56.0<br>☐ Pyrexia of unknown origin R50.9<br>☐ Prematurity and low birth weight (LBW) P07<br>☐ Urinary tract infection N39.0<br>☐ Neonatal jaundice P59.9<br>☐ Meningitis G03.9<br>☐ Rheumatic Fever I00<br>☐ Others (Specify)..	☐ Discharge with advice<br>☐ Discharge on request<br>☐ Discharge on risk bond (DORB)<br>Time & Date of discharge:<br>...<br>☐ Referred<br>Time & Date of Referral:<br>...<br>Place of referral: ....<br>☐ Absconded<br>☐ Death<br>Time & Date of Death<br>.......................<br>Cause of Death:<br>..................................<br>Has the MPDSR Death notification form been filled?<br>☐ Yes ☐ No	

Supplementary Table S2: Background characteristics of the government appointed nurses receiving training on inpatient register (N=176)

Characteristics	n	%
**Age**		
< 35 years	94	53
≥ 35 years	82	47
Missing	0	
**District**		
Kushtia	65	37
Dinajpur	111	63
Missing	0	
**Facility Type**		
District hospital	27	15
Sub-district hospital	149	85
Missing	0	
**Total (N)**	**176**	

Supplementary Table S3: Influence of different patient, and facility related factors on level of observed agreement between healthcare providers and study nurses regarding reporting different variables in the inpatient register, presented as kappa coefficient (N=4849)

	Treatment oxygen given	Treatment Inj. Gentamicin	Diagnosis- Severe Pneumonia	Refer
	Kappa	Kappa	Kappa	Kappa
**Patient related factors**				
**Age**				
0-28 days	0.28	0.74	0.74	0.74
29 days-11 months	0.41	0.76	0.66	0.66
12-59 months	0.54	0.84	0.71	0.71
**Sex**				
Male	0.46	0.78	0.70	0.70
Female	0.49	0.77	0.72	0.72
**Facility related factors**				
**Facility type**				
District hospital	0.36	0.73	0.70	0.77
Sub-district hospital	0.61	0.84	0.75	0.94
**District**				
Kushtia	0.46	0.76	0.72	0.79
Dinajpur	0.36	0.75	0.53	0.76
**Month of assessments**				
Month 1	0.39	0.72	0.69	0.80
Month 2	0.55	0.78	0.68	0.78
Month 3	0.48	0.82	0.75	0.79
**Total**	**0.47**	**0.77**	**0.71**	**0.79**
